# Images in Clinical Tropical Medicine: Lupus Vulgaris

**DOI:** 10.4269/ajtmh.21-0736

**Published:** 2021-09-13

**Authors:** Palaniappan Vijayasankar, Raveendran Premjith, Kaliaperumal Karthikeyan

**Affiliations:** Department of Dermatology, Venereology and Leprosy, Sri Manakula Vinayagar Medical College and Hospital, Pondicherry, India

**Figure 1.**
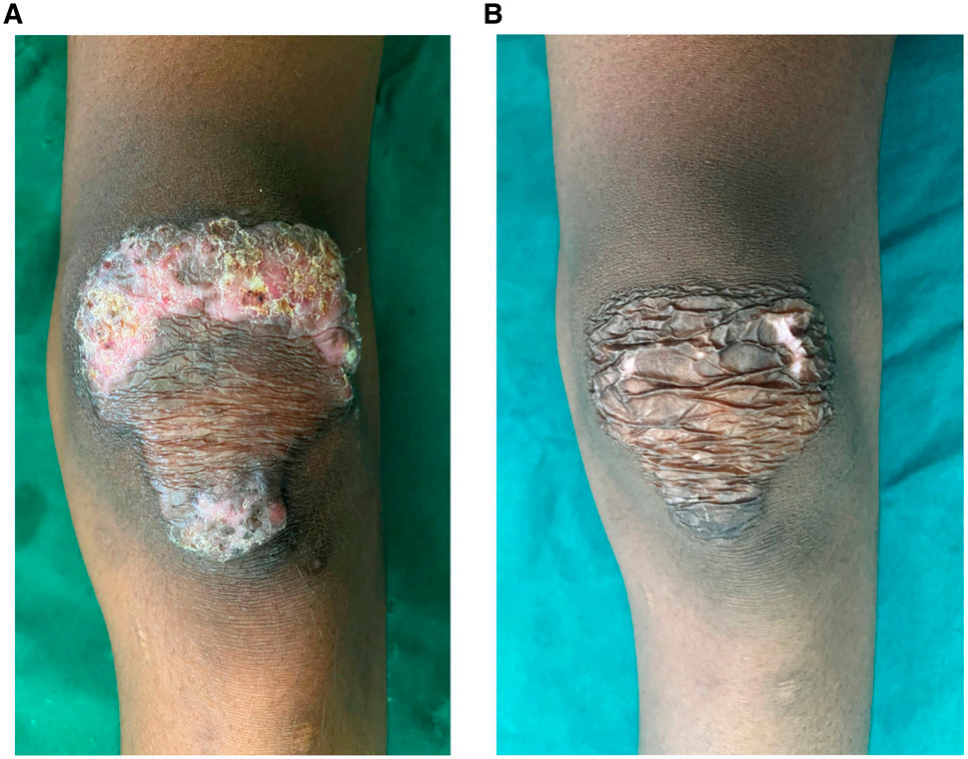
**(A)** A 7.25 × 6.25-cm pear-shaped, well-defined erythematous scaly plaque with crusting and areas of atrophy and scarring. (**B**) Lesion resolved, with wrinkled atrophic scarring after treatment. This figure appears in color at www.ajtmh.org.

**Figure 2.**
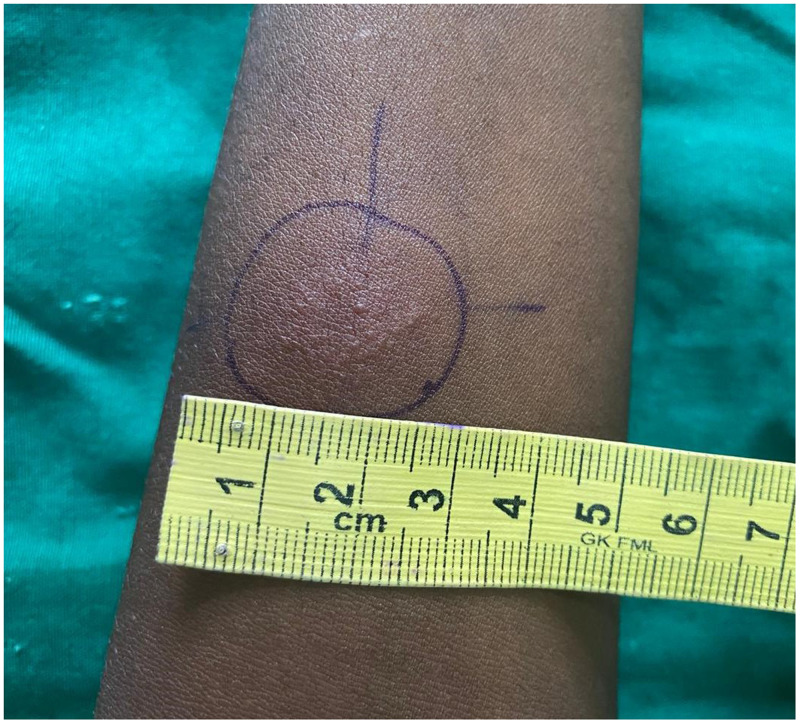
Positive tuberculin test. This figure appears in color at www.ajtmh.org.

**Figure 3.**
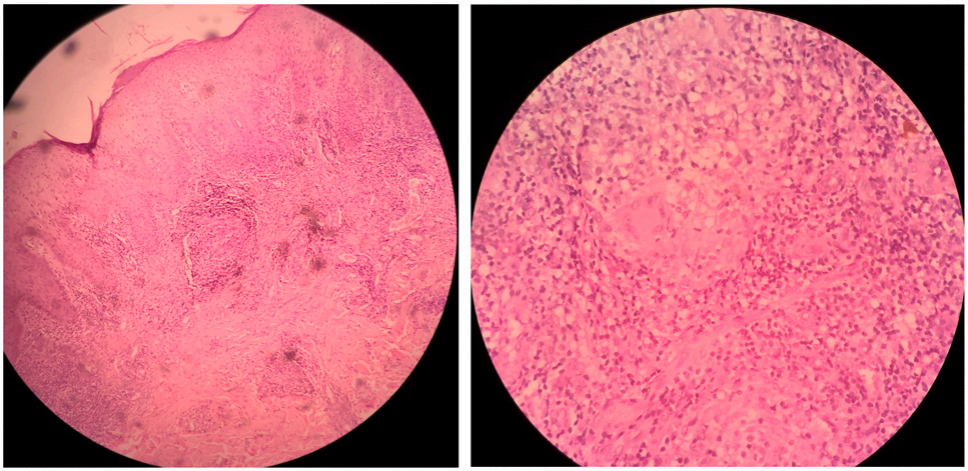
**(A)** Hematoxylin–eosin staining and low-power view shows multiple well-formed epithelioid granulomas with or without giant cells surrounded by lymphocytic infiltrates in the dermis. (**B**) Hematoxylin–eosin staining and high-power view shows granuloma with giant cells and lymphoplasmacytic infiltrates. This figure appears in color at www.ajtmh.org.

A 16-year-old Indian boy presented with an 8-year history of an asymptomatic, slowly progressive skin lesion over the left knee. Examination showed a single, well-defined erythematous scaly plaque with crusting, and areas of atrophy and scarring (Figure 1A). the general examination was normal. The patient’s past medical history and family history were not significant. A tuberculin skin test was strongly positive (Figure 2). Incisional biopsy revealed multiple well-formed epithelioid granulomas with or without giant cells surrounded by lymphocytic infiltrates in the dermis (Figure 3). Ziehl-Neelson staining of the tissue section, mycobacterial culture of a tissue specimen, and polymerase chain reaction for *Mycobacterium tuberculosis* were negative. Based on the clinicopathologic findings, a diagnosis of lupus vulgaris was made. The patient was treated with antituberculous therapy (rifampicin, isoniazid, pyrazinamide, and ethambutol) for 6 months. At the end of treatment, the lesion had healed well, leaving an atrophic, wrinkled scar (Figure 1B).

Lupus vulgaris is a chronic, progressive paucibacillary form of cutaneous tuberculosis. Criteria for diagnosis are variable. Tuberculin skin testing is usually positive.^1^ Analysis of a biopsy specimen should include histopathology, tissue smear, bacteriologic cultures, and polymerase chain reaction, but may be negative, as in this case of paucibacillary disease reminiscent of tuberculoid leprosy.^2^ Assessment for pulmonary and extrapulmonary tuberculosis should be done.^3^ Complications may occur, including secondary bacterial infections, mutilation, destruction, scarring, and joint contractures.^3^^,^^4^ Cutaneous tuberculosis generally responds well to antituberculous treatment.^2^
